# Effects of dexmedetomidine nasal spray combined with propofol for deep sedation in patients undergoing endoscopic retrograde cholangiopancreatography: a prospective randomized study

**DOI:** 10.3389/fmed.2026.1832298

**Published:** 2026-05-07

**Authors:** Jie Wu, Jiesong Liu, Xiaocao Xu, Kan He, Shiyou Wei, Tao Chen, Xiaohu Yang

**Affiliations:** 1 Department of Anesthesiology, Shanghai East Hospital, Tongji University School of Medicine, Shanghai, China; 2 Department of Clinical Laboratory, Longhua Hospital Affiliated to Shanghai University of Traditional Chinese Medicine, Shanghai, China; 3 Department of Anesthesiology, Shanghai Pulmonary Hospital, Tongji University School of Medicine, Shanghai, China

**Keywords:** deep sedation, dexmedetomidine, endoscopic retrograde cholangiopancreatography, nasal spray, propofol

## Abstract

**Background:**

Endoscopic retrograde cholangiopancreatography (ERCP) requires effective and safe deep sedation. Dexmedetomidine is a promising sedative in painless procedures. This study investigated the efficacy and safety of preoperative dexmedetomidine via nasal spray and conventional intravenous infusion for ERCP deep sedation.

**Methods:**

In this single-center, prospective randomized trial, 180 adult patients scheduled for ERCP were assigned to three groups: preoperative nasal spray (Group NS), intravenous pumping (Group IP), or control without dexmedetomidine (Group C). A propofol-based protocol was applied to all groups. Primary outcome was the incidence of intraoperative hypoxemia. Secondary outcomes included propofol consumption, hemodynamic stability, recovery profiles, and the incidence of other adverse events.

**Results:**

Compared to Group C, both Group NS and Group IP significantly reduced intraoperative hypoxemia (5.0% vs. 5.0% vs. 21.7%, *p* < 0.001), lowered propofol requirements, and improved postoperative recovery (shorter time to consciousness, lower agitation and pain scores). Group NS achieved comparable clinical benefits to Group IP but with significantly shorter anesthesia time (48.9 ± 5.2 min vs. 59.7 ± 6.4 min, *p* < 0.001) and higher endoscopist satisfaction (9 (9, 10) vs. 8 (7, 8), *p* < 0.001).

**Conclusion:**

Dexmedetomidine nasal spray combined with propofol is as effective and safe as the intravenous route for ERCP deep sedation, offering the additional advantages of shorter anesthesia duration and greater procedural convenience.

**Clinical trial registration:**

https://register.clinicaltrials.gov, identifier NCT07204106.

## 1 Introduction

Endoscopic retrograde cholangiopancreatography (ERCP) is an endoscopic technique for diagnosing and treating pancreatic and biliary diseases, serving as a crucial diagnostic and therapeutic modality for biliary and pancreatic disorders. The adoption of painless anesthesia has significantly improved patient tolerance and facilitated the performance of this invasive procedure. However, a substantial proportion of ERCP patients are elderly with poor physical conditions, which makes them more likely to experience cardiopulmonary suppression (1, 2). This necessitates a delicate balance to ensure stable respiratory and circulatory functions while simultaneously achieving adequate anesthesia for procedural comfort and success.

Currently, deep sedation with monitored anesthesia and general anesthesia with tracheal intubation are the two primary anesthesia methods for ERCP procedures (3, 4). Deep sedation, a technique that obviates the need for endotracheal intubation, is characterized by its minimal physiological perturbation and potential for rapid recovery, which has contributed to its increasing utilization in such procedures (5, 6). Propofol is widely used for sedation during ERCP due to its rapid onset and recovery profile (6, 7). However, its use may be associated with a higher risk of respiratory depression and hypotension (8). Therefore, other sedatives are often combined with propofol to reduce its required dosage and mitigate associated risks.

Dexmedetomidine, a selective α2-adrenergic receptor agonist, demonstrates sedative, anti-anxiety, anti-sympathetic, and analgesic effects with minimal respiratory depression. In recent years, its application in ERCP has gained increasing attention owing to its sufficient sedative effects and less cardiopulmonary inhibition (9, 10). However, an initial bolus is often required a period of 10 min for intravenous pumping, which significantly prolongs the anesthesia procedure and impacts the efficiency. Nasal spray has shown comparable efficacy, making it a preferred choice for sedation (11, 12). Therefore, the study team hypothesized the convenience and safety profile of nasal spray of dexmedetomidine further facilitate sedation for ERCP. This study aimed to investigates the safety and effectiveness of preoperative nasal dexmedetomidine inhalation in deep sedation-based ERCP procedure.

## 2 Methods

This study was a parallel-designed randomized study performed at Shanghai East Hospital, China. The study protocol was approved by the ethics committee of Shanghai East Hospital (Ethics No.: 2025YS-176), with all patients signing informed consent forms before grouping. On September 17, 2025, this study was registered at the clinicaltrials.gov (Registration No.: NCT07204106). The authors declare that the study was performed in accordance with the Declaration of Helsinki and reported in line with the CONSORT 2025 guideline (13, 14).

### 2.1 Inclusion and exclusion criteria

Participants were adult patients aged 18–80 years undergoing ERCP at Shanghai East Hospital between October and November 2025. Eligible individuals had a body mass index (BMI) of 18–30 kg/m^2^ and an American Society of Anesthesiologists (ASA) physical status classification of I to III. Exclusion criteria were as follows: (1) explicit refusal to participate; (2) respiratory distress defined as a Modified Marquardt score of Grade IV; (3) anemia (hemoglobin <90 g/L) or thrombocytopenia (platelet count < 80 × 10^9^/L); (4) significant liver or renal dysfunction; (5) a history of nasal trauma or nasal septum deviation; (6) known hypersensitivity to dexmedetomidine, opioids, propofol, rocuronium, or any of their components; and (7) a history of abnormal recovery from previous anesthesia or surgery.

Participants could be withdrawn from the study at any time under the following circumstances: (1) voluntary withdrawal of consent by the participant or their legal representative; (2) development of severe adverse events (SAEs) or intolerable side effects related to the study intervention, as judged by the investigator; (3) occurrence of significant intraoperative hemodynamic instability or respiratory compromise necessitating emergency airway management; (4) substantial deviation from the protocol by the investigator or participant that could affect the study outcomes; (5) decision by the investigator that continued participation is no longer in the best interest of the participant; or (6) administrative reasons, such as loss to follow-up or the participant becoming unavailable.

### 2.2 Randomization and blinding

Patients were randomly assigned to one of three groups with a ratio of 1:1:1 using a computer-generated random number table: preoperative nasal spray group (Group NS), conventional intravenous pumping group (Group IP) or control group (Group C). Randomization was performed by an independent statistician not involved in patient recruitment or data collection. The allocation sequence was concealed in sequentially numbered, opaque, sealed envelopes to ensure allocation concealment. The anesthesiologist responsible for patient recruitment and anesthesia administration was unaware of the treatment assignments until after the patient had been enrolled and the envelope was opened. In addition, placebos were used to maintain allocation concealment.

To minimize bias, the anesthesiologist who collected data during study was not involved in the randomization or blinding process. Post-anesthesia care unit (PACU) evaluations and postoperative follow-up assessments were conducted by a different, independent anesthesiologist who was also blinded to the treatment group allocation. Crucially, both the patients and the data collectors (intraoperative and postoperative) were unaware of the specific sedation regimen administered.

### 2.3 Interventions and groups

Group NS (dexmedetomidine nasal spray + intravenous saline infusion): Thirty minutes prior to the procedure, patients received intranasal dexmedetomidine spray (Hengrui Pharmaceuticals, Lianyungang City, Jiangsu Province, China) in the anesthesia preparation room. Considering the effects of potential for drug synergism, the dose was 1 μg/kg (15). The total dosage was 50 to 100 μg (16). Saline was administered intravenously over 10 min. For induction, intravenous sufentanil (0.1 μg/kg; Humanwell Healthcare, Wuhan City, Hubei Province, China) was administered, followed by propofol (1.5–2.0 mg/kg; Fresenius Kabi (Beijing) Pharmaceuticals, Beijing City, China) injected over at least 30 s. Propofol was administered as a continuous IV infusion at an initial rate of 4–12 mg/kg/h for maintenance.

Group IP (saline nasal spray + intravenous dexmedetomidine): Thirty minutes prior to the procedure, patients received intranasal saline spray in the anesthesia preparation room. For induction, intravenous dexmedetomidine (0.5 μg/kg; Yangtze River Pharmaceutical Group, Taizhou City, Jiangsu Province, China) was administered over 10 min. Immediately following the dexmedetomidine loading dose, sufentanil and propofol were administered using the same protocol as in Group NS.

Group C (saline nasal spray + intravenous saline infusion): Anesthesia was induced and maintained using sufentanil and propofol, following the identical protocol described above for the other groups. Saline nasal spray and intravenous saline infusion were administered as the intervention groups.

### 2.4 Anesthesia procedure

All patients were required to fast for at least 8 h and abstain from clear liquids for 4 h prior to the procedure. Standard ASA monitors were applied immediately, including non-invasive blood pressure (NIBP), heart rate (HR), electrocardiogram (ECG), pulse oximetry (SpO₂), and bispectral index (BIS). Patients were then positioned in the left lateral decubitus position and pre-oxygenated with 100% oxygen via a nasal cannula at 5 L/min. Anesthesia induction and maintenance protocols were according to the specific groups. The infusion rate was titrated based on BIS values to maintain a target range of 40–60. If the patient exhibited signs of inadequate sedation, evidenced by the Modified Observer’s Assessment of Alertness/Sedation Scale (MOAA/S) during the procedure, a bolus of propofol (50% of the initial induction dose) was administered. The interval between supplemental boluses was at least 3 min.

Intraoperative management of adverse events was standardized. For hypoxemia (SpO₂ < 90% for >10 s), initial management included increasing the oxygen flow rate by 2 L/min (maximum 10 L/min), jaw thrust maneuver, and/or insertion of a nasopharyngeal airway. If these measures were ineffective, assisted ventilation was employed. For intraoperative hypotension (systolic blood pressure < 90 mmHg or a decrease in mean arterial pressure > 20% from baseline for >1 min), intravenous phenylephrine (4–12 μg bolus) was administered. For bradycardia (heart rate <50 beats per minute for >1 min), intravenous atropine (0.3–0.5 mg) was injected.

At the conclusion of ERCP, propofol administration was discontinued. The patients were sent to the Post-Anesthesia Care Unit (PACU) until MOAA/S score was ≥ 4. The Aldrete score was calculated every five minutes. Patients were transferred from the PACU to the ward when they achieved an Aldrete score of ≥ 9 for three consecutive measurements.

### 2.5 Outcomes

The primary observation indicator was the incidence of intraoperative hypoxemia (defined as SpO_2_ < 90% for over 10 s). The secondary observation indicators included propofol dose, anesthesia time, circulatory stability, intraoperative hypotension, bradycardia and arrhythmias, airway interventions, time to consciousness, agitation and sedation levels, pain score, and postoperative adverse events. Anesthesia time was defined as the duration from the start of anesthesia induction to the completion of emergence. For Group IP, this included the 10-min intravenous dexmedetomidine loading dose. The 30-min preoperative nasal administration in Group NS was performed in the preparation room and was not included in anesthesia time. SBP, DBP, mean arterial pressure (MAP), and heart rate (HR) were recorded at the following time points: before anesthesia (T0), post-induction (T1), endoscopic esophagoscope insertion (T2), duodenal papilla intubation (T3), endoscope removal (T4), and patient awakening (T5).

### 2.6 Statistical analysis

The sample size determination was based on the preliminary observation. The incidences of hypoxemia were 40, 25, and 15% in Group C, Group NS, and Group IP, respectively. Given an *α* of 0.05, a *β* of 0.8, and a dropout rate of 5%, 60 patients were needed in each group.

Statistical analyses were performed using SPSS 31.0 software (IBM Corp., Armonk, NY, United States). Continuous data following a normal distribution were expressed as mean ± standard deviation (x̄±SD), and inter-group comparisons were conducted using one-way analysis of variance (ANOVA). If a significant overall difference was detected (*p* < 0.05), post-hoc pairwise comparisons were performed using the Bonferroni correction to adjust for type I error. Hemodynamic data at each time point were compared among groups using repeated-measures ANOVA with Bonferroni post-test. For continuous data with a non-normal distribution, median (M) and interquartile range (IQR) were used for description, and the Kruskal–Wallis H test was applied for inter-group comparisons. If significant differences were identified, pairwise comparisons were performed using the Mann–Whitney U test with Bonferroni correction. Categorical data were presented as counts and percentages (*n*, %), and comparisons between groups were performed using the chi-square (χ^2^) test or Fisher’s exact test, depending on the expected cell frequencies. A two-tailed *p* value <0.05 was considered statistically significant.

## 3 Results

**Figure 1:**
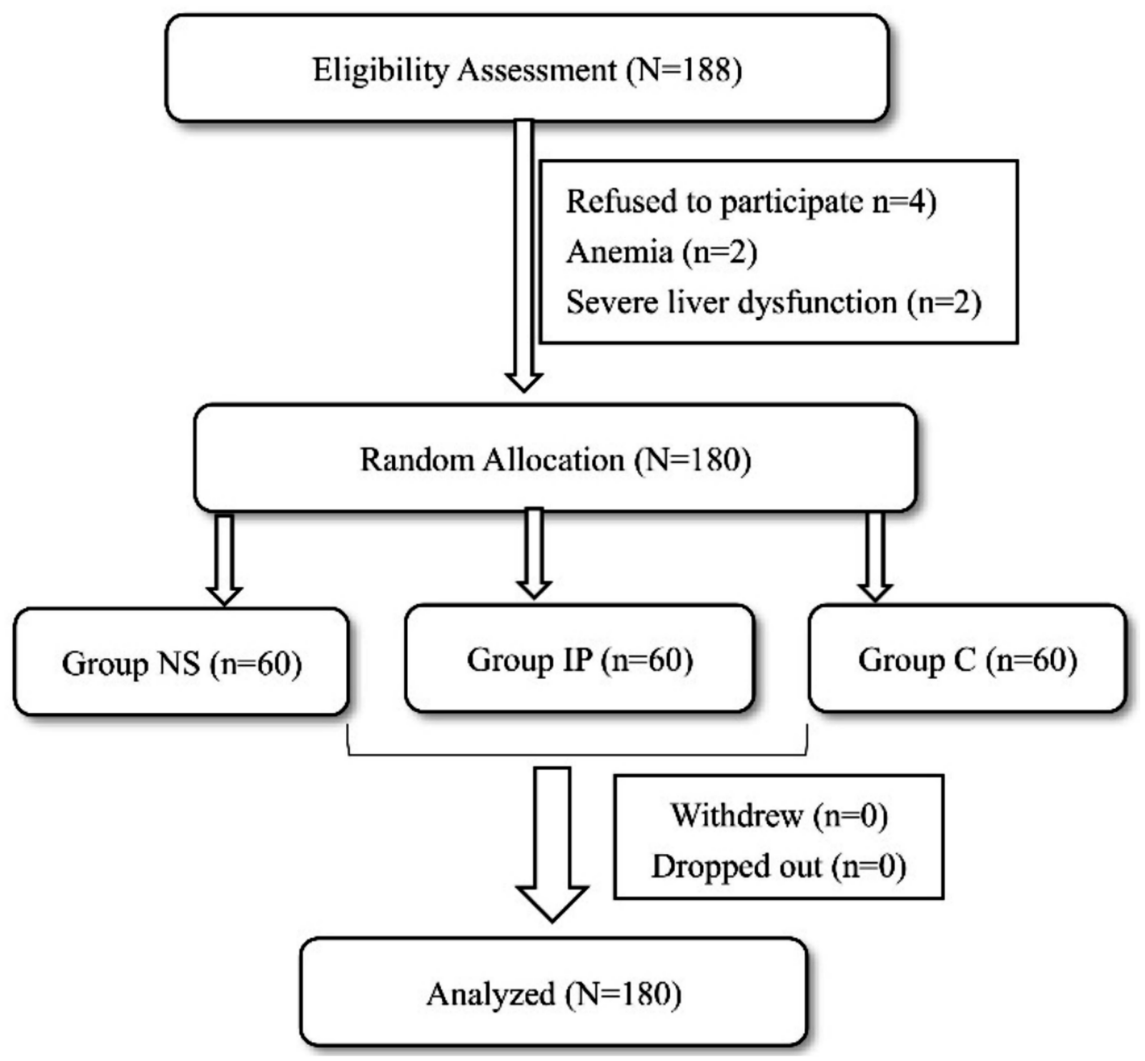
Study flowchart. NS, nasal spray; IP, intravenous pumping; C, control.

The study flowchart was shown in Figure 1. A total of 188 patients initially met the study’s inclusion criteria. Among these, 8 patients were excluded due to failure to meet the exclusion criteria (see Figure 1 for details). Finally, 180 eligible patients were enrolled and evenly allocated to three groups for the final analysis, with 60 patients in each group.

As shown in Table 1, there were no statistically significant differences in baseline characteristics among the three groups.

**Table 1:** General information of the patients.

Variables	Group NS (*n* = 60)	Group IP (*n* = 60)	Group C (*n* = 60)	*p* value
Age (year)	46.4 ± 14.2	42.3 ± 15.4	47.1 ± 16.3	0.18
Height (cm)	168.8 ± 6.8	168.7 ± 7.6	168.4 ± 7.6	0.97
Weight (kg)	66.1 ± 8.9	66.4 ± 9.1	65.9 ± 9.4	0.95
BMI $\left(kg/m^{2}\right)$	23.2 ± 2.8	23.3 ± 2.6	23.2 ± 2.7	0.95
Gender, Male, *n* (%)	27 (45.0)	29 (48.3)	30 (50.0)	0.86
Ethnicity, Han Chinese, *n* (%)	60 (100)	60 (100)	60 (100)	1
ASA (*n*, %)				0.89
I	21 (35)	22 (37)	20 (33)	
II	32 (53)	34 (57)	35 (58)	
III	7 (12)	4 (7)	5 (8)	

The incidence of intraoperative hypoxemia was significantly lower in Group NS [5.0%; (95% CI: 1.7–13.7%)] and Group IP [5.0%; (95% CI: 1.7–13.7%)] compared with Group C [21.7%; (95% CI: 13.1–33.6%)] (*p* < 0.001) (Table 2). Similarly, the rate of airway interventions (including jaw thrust and nasopharyngeal airway intubation) was markedly lower in Group NS (3.3%) and Group IP (5.0%) than in Group C (31.6%) (*p* < 0.001) (Table 2). The incidence of intraoperative hypotension also showed significant differences: Group NS (3.3%) and Group IP (6.7%) had lower rates than Group C (23.3%) (*p* < 0.001) (Table 2). In contrast, bradycardia was more prevalent in Group NS (20.0%) and Group IP (28.3%) compared with Group C (5.0%) (*p* < 0.01) (Table 2). No arrhythmia was reported in any of the three groups.

**Table 2:** Comparison of intraoperative indicators.

Variables	Group NS (*n* = 60)	Group IP (*n* = 60)	Group C (*n* = 60)	*p* value
ERCP time (min)	40.4 ± 6.2 (95% CI: 38.8–42.0)	41.1 ± 5.6 (95%CI: 39.7–42.5)	39.6 ± 6.1 (95%CI: 38.0–41.2)	0.36
Anesthesia time (min)	48.9 ± 5.2 (95% CI: 47.6–50.2)^b^	59.7 ± 6.4 (95%CI: 58.0–61.4)^a^	51.2 ± 7.6 (95%CI: 49.2–53.2)^b^	<0.001
Dose of propofol (mg)	310.9 ± 35.7 (95% CI: 301.7–320.1)^a^	302.9 ± 46.6 (95%CI: 290.9–314.9)^a^	373.7 ± 38.9 (95%CI: 363.7–383.7)	<0.001
Hypoxemia, *n* (%)	3 (5.0) (95% CI: 1.7–13.7%)^a^	3 (5.0) (95%CI: 1.7–13.7)^a^	13 (21.7) (95%CI: 13.1–33.6)	<0.001
Airway intervention	2 (3.3) (95% CI: 0.8–11.3%)^a^	3 (5.0) (95%CI: 1.7–13.7)^a^	19 (31.6) (95%CI: 21.0–44.7)	<0.001
Jaw thrust, *n* (%)	2 (3.3) (95% CI: 0.8–11.3%)	2 (3.3) (95%CI: 0.8–11.3)	10 (16.6) (95%CI: 9.0–28.1)	
Nasopharyngeal airway intubation, *n* (%)	0 (0) (95% CI: 0–6.0%)	1 (1.7) (95%CI: 0.2–9.0)	9 (15.0) (95%CI: 7.8–26.0)	
Hypotension, *n* (%)	2 (3.3) (95% CI: 0.8–11.3%)^a^	4 (6.7) (95%CI: 2.2–16.2)^a^	14 (23.3) (95%CI: 14.2–35.2)	<0.001
Bradycardia, *n* (%)	12 (20.0) (95%CI: 11.3–32.2)^a^	17 (28.3) (95%CI: 17.9–41.4)^a^	3 (5.0) (95%CI: 1.7–13.7)	<0.01
Arrhythmia, *n* (%)	0 (0) (95%CI: 0.0–6.0)	0 (0) (95%CI: 0.0–6.0)	0 (0) (95%CI: 0.0–6.0)	1

a *p* < 0.05 compared with Group C.

b *p* < 0.05 compared with Group IP.

Continuous variables (ERCP time, anesthesia time, propofol dose) were compared using one-way ANOVA with Bonferroni post-test. Categorical variables (hypoxemia, airway intervention, jaw thrust, nasopharyngeal airway intubation, hypotension, bradycardia, arrhythmia) were compared using χ^2^ test or Fisher’s exact test.

The ERCP time was comparable among the three groups (*p* = 0.36), with no significant differences observed (Table 2). However, the anesthesia time differed significantly across groups (*p* < 0.001): Group IP had the longest anesthesia time (59.7 ± 6.4 min; 95% CI: 47.6–50.2 min), which was significantly longer than that in Group NS (48.9 ± 5.2 min; 95% CI: 58.0–61.4 min) and Group C (51.2 ± 7.6 min; 95% CI: 49.2–53.2 min) (Table 2).

In terms of anesthetic dosage, the total propofol dose in Group NS (310.9 ± 35.7 mg; 95% CI: 301.7–320.1 mg) and Group IP (302.9 ± 46.6 mg; 95% CI: 290.9–314.9 mg) was significantly lower than that in Group C (373.7 ± 38.9 mg; 95% CI: 363.7–383.7 mg) (*p* < 0.001), while no significant difference was found between Group NS and Group IP (Table 2).

**Figure 2:**
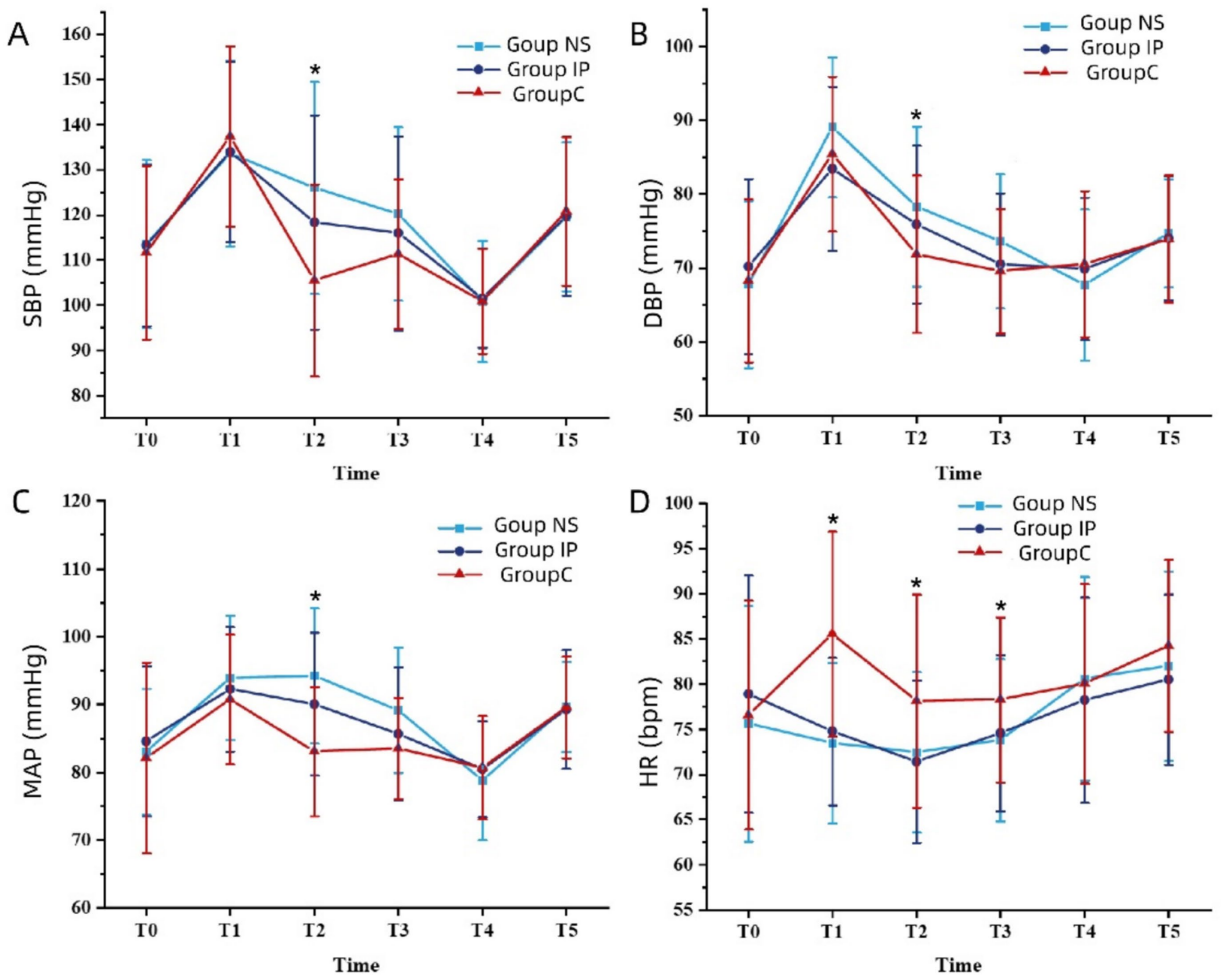
Differences in systolic blood pressure (SBP) **(A)**, diastolic blood pressure (DBP) **(B)**, mean arterial pressure (MAP) **(C)**, and heart rate (HR) **(D)** among the groups. *, *p* < 0.05. Data at each time point were compared among groups using repeated-measures ANOVA with Bonferroni post-test.

Figure 2 shows the intraoperative changes of SBP, DBP, MAP and HR in the three groups. At T1 (post-induction), the SBP, DBP and MAP of Group NS were significantly higher than those of Group C, while the HR was significantly lower than that of Group C (*p* < 0.05); during T2–T3 (intraoperative stimulation), the HR of Group NS and Group IP were consistently lower than that of Group C, with statistical differences (*p* < 0.05). There were no significant differences in the four hemodynamic indicators among the three groups at other time points.

Postoperative outcomes were presented in Table 3. The time to consciousness was significantly shorter in Group NS (6.7 ± 2.2 min; 95% CI: 6.1–7.3 min) and Group IP (7.3 ± 3.0 min; 95% CI: 6.5–8.1) than in Group C (9.8 ± 2.1 min; 95% CI: 9.3–10.3 min) (*p* < 0.001), while the total recovery time showed no significant difference among the three groups (*p* = 0.31). Regarding postoperative comfort, the Richmond Agitation-Sedation Scale (RSAS) score was significantly lower in Group NS (1.7 ± 1.4) and Group IP (1.7 ± 1.3) compared with Group C (3.4 ± 1.0) (*p* < 0.001), indicating less postoperative agitation in the two dexmedetomidine groups. The Visual Analog Scale (VAS) pain score was also significantly lower in Group NS (2.4 ± 2.1) and Group IP (3.0 ± 2.6) than in Group C (5.5 ± 2.8) (*p* < 0.001), reflecting better postoperative analgesic effects. For postoperative adverse events (dizziness, drowsiness, nausea, and vomiting), there were no statistically significant differences between the three groups (all *p* > 0.05). Only one patient in Group C reported vomiting, while the incidence of other mild adverse events was low and comparable across groups.

**Table 3:** Comparison of recovery indicators and postoperative adverse events.

	Group NS (*n* = 60)	Group IP (*n* = 60)	Group C (*n* = 60)	*p* value
Time to consciousness (min)	6.7 ± 2.2 (95%CI: 6.1–7.3)^a^	7.3 ± 3.0 (95%CI: 6.5–8.1)^a^	9.8 ± 2.1 (95%CI: 9.3–10.3)	<0.001
Recovery time (min)	16.0 ± 5.3 (95%CI: 14.6–17.4)	15.7 ± 4.8 (95%CI: 14.5–16.9)	15.1 ± 4.4 (95%CI: 14.0–16.2)	0.31
RSAS score	1.7 ± 1.4 (95%CI: 1.3–2.1)^a^	1.7 ± 1.3 (95%CI: 1.4–2.0)^a^	3.4 ± 1.0 (95%CI: 3.1–3.7)	<0.001
VAS score	2.4 ± 2.1 (95%CI: 1.8–3.0)^a^	3.0 ± 2.6 (95%CI: 2.3–3.7)^a^	5.5 ± 2.8 (95%CI: 4.8–6.2)	<0.001
Dizziness, *n* (%)	2 (3.3) (95%CI: 0.8–11.3)	3 (5.0) (95%CI: 1.7–13.7)	5 (8.3) (95%CI: 3.1–18.1)	0.48
Drowsiness, *n* (%)	6 (10.0) (95%CI: 4.3–20.1)	8 (13.3) (95%CI: 6.4–24.4)	8 (13.3) (95%CI: 6.4–24.4)	0.81
Nausea, *n* (%)	4 (6.7) (95%CI: 2.2–16.2)	3 (5.0) (95%CI: 1.7–13.7)	7 (11.7) (95%CI: 5.1–22.6)	0.38
Vomiting *n* (%)	0 (0) (95%CI: 0.0–6.0)	0 (0) (95%CI: 0.0–6.0)	1 (1.7) (95%CI: 0.2–9.0)	1
Satisfaction of patients	9 (9, 10)	9 (9, 9)	9 (9, 9)	0.45
Satisfaction of endoscopists	9 (9, 10)^b^	8 (7, 8)	9 (9, 9)^b^	<0.001

a *p* < 0.05 compared with Group C.

b *p* < 0.05 compared with Group IP.

RSAS, Richmond Agitation-Sedation Scale; VAS, Visual Analog Scale. Continuous variables (time to consciousness, recovery time, RSAS score, VAS score) were compared using one-way ANOVA with Bonferroni post-test. Categorical variables (dizziness, drowsiness, nausea, vomiting) were compared using χ^2^ test or Fisher’s exact test. Satisfaction scores were compared using Kruskal–Wallis H test.

The satisfaction level of the patients on the perioperative experience was comparable among the groups, while the endoscopists thought that the induction time was too long in the Group IP and gave the lowest scores (Table 3).

## 4 Discussion

This randomized parallel-designed trial evaluated the efficacy and safety of dexmedetomidine nasal spray in elective ERCP procedures. The results demonstrated that both nasal spray and intravenous dexmedetomidine significantly decreased hypoxemia incidence during ERCP, reduced propofol dosage, alleviated intraoperative blood pressure and heart rate fluctuations, and improved postoperative recovery quality compared to the standard propofol-sufentanil regimen. However, nasal spray significantly shortened the duration of anesthesia induction and improved the satisfaction of the endoscopists in comparison with intravenous pumping.

ERCP examination is a discomfortable and painful procedure. Conscious sedation cannot completely relieve the discomfort, especially for young patients, which makes deep sedation with anesthetics a favored choice in clinical practice (17, 18). Dexmedetomidine, a potent selective α2-adrenergic receptor agonist, demonstrates sedative, analgesic, and anti-sympathetic properties along with anti-inflammatory and organ-protective effects (19). In recent years, dexmedetomidine has been widely used in anesthesia procedures (20–22).

In the present study, the combination of dexmedetomidine with propofol demonstrated distinct clinical benefits over propofol-sufentanil monotherapy. First, it significantly reduced anesthetic consumption and related adverse events. Groups NS and IP required 16.8 and 19.0% less propofol than Group C, respectively, which is consistent with previous studies showing that dexmedetomidine’s sedative and analgesic effects reduce the demand for other anesthetics (9). Correspondingly, the incidence of intraoperative hypoxemia in the two dexmedetomidine groups (5.0% each) was drastically lower than in Group C (21.7%), and the rate of airway interventions (3.3 and 13.3% vs. 31.6%) and hypotension (3.3 and 6.7% vs. 23.3%) were also significantly reduced. This is attributed to dexmedetomidine’s minimal respiratory depression effect, which mitigates the respiratory suppression caused by propofol (23). Second, it improved postoperative recovery quality. The time to consciousness was 31.6 and 25.5% shorter in Groups NS and IP than in Group C, and the RSAS and VAS scores were significantly lower, indicating less postoperative agitation and better analgesia. This aligns with a latest meta-analysis enrolled nearly 6,000 patients (24). Notably, postoperative adverse events such as dizziness and nausea were comparable across groups, confirming the safety of adding dexmedetomidine. Finally, it enhanced intraoperative hemodynamic stability. During key stimulation points (esophagoscope insertion and papilla intubation), Groups NS and IP maintained more stable heart rates than Group C, reflecting the ability of dexmedetomidine to suppress sympathetic activation (25). It should be acknowledged that the incidence of bradycardia was higher in the dexmedetomidine groups (20.0% in NS group and 28.3% in IP group) than in the control group (5.0%). This finding is consistent with the known pharmacology of dexmedetomidine, which reduces central sympathetic outflow and increases vagal tone (26). However, all bradycardic events were transient, asymptomatic, and clinically manageable with either observation or low-dose atropine; no episode led to serious hypotension, hemodynamic collapse, or procedure interruption. These results demonstrate a favorable clinical trade-off: dexmedetomidine substantially decreased hypoxemia and hypotension at the cost of an increased but benign and manageable rate of bradycardia. Therefore, the overall perioperative respiratory–circulatory profile was safer and more stable in the dexmedetomidine groups.

Compared to intravenous dexmedetomidine, the nasal spray formulation offers unique clinical value in addition to equivalent sedative and safety profiles. Operationally, nasal spray is non-invasive and easy to administer. Before the procedure, the medication can be administered in the anesthesia preparation room, which is highly safe in clinical practice (11, 27). After nasal administration, dexmedetomidine reaches peak plasma concentration within 0.5–1 h (16). Consistent with this pharmacokinetic profile, the present study demonstrated the superior effects of dexmedetomidine nasal spray 30 min before ERCP as previously mentioned. In particular, nasal spray is associated with a shorter anesthesia time (48.9 ± 5.2 min vs. 59.7 ± 6.4 min in Group IP). This difference is originated from the medication method of intravenous dexmedetomidine, namely a 10-min induction time. Ten minutes is vital in practice and can impact the satisfaction of the medical staff. The endoscopists gave the lowest scores to intravenous dexmedetomidine in spite of its advantages.

Several limitations should be acknowledged. First, this was a single-center study with a relatively homogeneous patient population, which may limit the generalizability of the results to diverse groups such as elderly patients over 80 years old or those with severe comorbidities. Second, this study did not monitor dexmedetomidine plasma concentrations, so the correlation between drug levels and clinical outcomes remains unclear. Third, subgroup analyses stratified by age, comorbidities, and ASA physical status were not performed. Considering the relatively limited sample size, stratified grouping would reduce statistical power and affect the reliability of the results. Since ERCP patients are generally elderly and accompanied by multiple underlying diseases and high ASA grades, further large-sample studies are required to verify the findings in high-risk subgroups. Fourth, plasma dexmedetomidine concentrations were not monitored. Nasal absorption is susceptible to individual variations in mucosal perfusion and physiological status. Although a standardized 30-min absorption interval was applied, inter-individual differences in drug uptake could not be quantified. Further studies with plasma drug detection are warranted. In addition, the follow-up period was limited to the immediate postoperative period, and long-term outcomes such as delayed delirium or quality of life were not evaluated.

## 5 Conclusion

Both nasal spray and intravenous dexmedetomidine combined with propofol are safe and effective for ERCP deep sedation, reducing anesthetic dosage, respiratory complications, and postoperative discomfort. The nasal spray formulation, with its time-saving property and operational convenience, is a promising alternative for ERCP. Future multi-center studies with larger sample sizes and longer follow-up are warranted to validate these findings and explore optimal doses for specific populations.

## Data Availability

The raw data supporting the conclusions of this article will be made available by the authors, without undue reservation.
